# Comparison of the accuracy of virtual and direct bonding of orthodontic accessories

**DOI:** 10.1590/2177-6709.24.4.046-053.oar

**Published:** 2019

**Authors:** Natalice Sousa de Oliveira, Bruno Frazão Gribel, Leniana Santos Neves, Elizabeth Maria Bastos Lages, Soraia Macari, Henrique Pretti

**Affiliations:** 1 Universidade Federal de Minas Gerais, Faculdade de Odontologia, Departamento de Odontopediatria e Ortodontia, Divisão de Odontopediatria (Belo Horizonte/MG, Brazil).; 2 Private practice (Belo Horizonte/MG, Brazil).

**Keywords:** Accuracy, Orthodontic brackets, Direct bonding, Indirect bonding

## Abstract

**Introduction:**

Conventional direct and indirect bonding techniques fail to obtain the ideal bracket position.

**Objective:**

To compare the accuracy of virtual and conventional direct bonding of orthodontic accessories.

**Methods:**

A single virtual configuration (dental mannequin with Class I malocclusion) served as basis for generating the reference model (treated virtually) and the intervention models (10 digital models and 10 solid models, obtained by means of prototyping). A total of 560 teeth were then equally distributed between a group of orthodontists (Group I, direct bonding; and Group II, virtual bonding), working in two different time intervals. The individual positions of the accessories were measured after three-dimensional superimposition with customized software. The Student’s-t test for paired samples, and Chi-square tests were used for statistical analysis, both at the level of significance of 5%.

**Results:**

In comparison of the errors in raw values, there were significant differences only in the vertical (*p*< 0.001) and horizontal dimensions (*p*< 0.001). Considering the groups of ranges by clinical limits of the deviations, these differences were significant in the three dimensions, vertical (*p*< 0.001), horizontal (*p*= 0.044) and angular (*p*= 0.044).

**Conclusion:**

Virtual bonding made it possible to obtain more precise/accurate positioning of the orthodontic accessories. The potential accuracy of this method brings new perspectives to refining the indirect bonding protocols.

## RESUMO

**Introdução:**

técnicas convencionais, diretas ou indiretas, de colagem de acessórios ortodônticos falham em obter a posição ideal desses.

**Objetivo:**

comparar a acurácia da colagem virtual e da colagem direta de acessórios ortodônticos por vestibular.

**Métodos:**

uma configuração virtual única (modelo de manequim com Classe I dentária) serviu como base para a geração do modelo de referência (tratado virtualmente) e dos modelos de intervenção (10 modelos digitais e 10 modelos sólidos, obtidos por prototipagem). Quinhentos e sessenta dentes foram, então, igualmente distribuídos, em dois tempos distintos, entre um grupo de ortodontistas (Grupo I: colagem direta; Grupo II: colagem virtual). As posições individuais dos acessórios foram aferidas após a sobreposição tridimensional de modelos, em *software* customizado. O teste*t* de Student para amostras pareadas e o teste Qui-quadrado foram usados para a análise estatística, ambos com nível de significância de 5%.

**Resultados:**

na comparação dos erros, em valores brutos, houve diferenças significativas apenas nas dimensões vertical (*p*< 0,001) e horizontal (*p* < 0,001). Considerando-se o agrupamento de faixas por limites clínicos dos desvios, essas diferenças foram significativas nas três dimensões: vertical (*p* < 0,001), horizontal (*p* = 0,044) e angular (*p* = 0,044).

**Conclusão:**

a colagem virtual possibilitou um posicionamento mais preciso dos acessórios ortodônticos. Seu potencial de precisão traz novas perspectivas ao refinamento dos protocolos indiretos.

## INTRODUCTION

The precise positioning of bonded accessories is considered one of the most challenging aspects for optimizing orthodontic treatment.^1^ ^,^ ^2^ The correct position potentiates, to a large extent, the expression of pre-adjusted appliances, and minimizes the need for additional interventions in the archwires, or re-bonding accessories.^3^ Studies have demonstrated that both the traditional direct and indirect bonding techniques fail to attain accuracy,^4^ ^,^ ^5^ although the latter is more consistent.^6^ ^,^ ^7^

In an endeavor to minimize human error during this critical stage of executing the treatment plan, there have been a growing number of researches directed towards refining the indirect bonding protocols, particularly after incorporating CAD/CAM technology into these process.^8^ ^,^ ^9^ Over the last few decades, different commercial systems have been made available, including the use of multifunctional platforms that have advanced technologies. As regards treatment planning and the laboratory stage, the digital systems have not been shown to guarantee accuracy.^10^ The traditional factors of imprecision,^11,12,13^ have been added to the limitations inherent to orthodontic software programs and the significant professional learning curve. 

At present, virtual bonding services are made available both for bonding directly in malocclusion, or post-setup.^7^ Although the second option increases the chances of success, the setup involves an additional cost and demands training for using software.

Considering that in clinical practice a large part of the work of bonding is performed without setup, previously measuring the quality of virtual bonding in this type of approach could contribute to enhancing the indirect method, and favoring adhesion to the digital systems, rather than using the direct technique. Moreover, no study evaluating the accuracy of digital bonding prior to guided bonding was found, or even comparing the precision of this bonding with the direct procedure. Thus, the aim of this study was to compare the accuracy of bracket placement between virtual and direct bonding procedures.

## MATERIAL AND METHODS

This was a prospective *in vitro* study, with a representative sample, in which orthodontists performed orthodontic accessory bonding using direct or indirect virtual bonding procedures, in models with identical type of malocclusion. For the sample size calculation, the findings of a previous clinical trial^14^ were considered, which pointed out a mean error of 0.26 mm, with standard deviation of 0.46, in the vertical positioning. For the comparison between two groups, at a level of significance of 5%, power of the study of 80% and presuming a clinical difference of 50% to be detected between the techniques, a total of 244 teeth and 9 participants would be needed for each intervention group.^15^ The sample size was enlarged to 280 teeth, with 10 participants per group, considering losses of 20%.

For the purpose of obtaining identical models regarding the pattern of malocclusion, a single dental mannequin (Dent-Art, São Paulo, Brazil), in normal occlusion, with complete dentition except for the third molars, was digitized (bench scanner - Scanner Ultrafast Optical Sectioning^™^ - Trios^®^ Orthodontic). After this, segmentation of teeth was performed (3 Shape software, OrthoAnalyzer^™^ module). Then, the specific positional changes were incorporated into multiple units (12 teeth, in the horizontal component; 6, in the angulations; 10, in the three dimensions, and 6 remained aligned and leveled). The resultant configuration (Class I malocclusion of the teeth, with slight/moderate crowding) served as basis for obtaining the reference model and intervention models (generation of 10 sets of digital models, and 10 sets of solid models, obtained by prototyping) (Eden 500 printer, Stratasys, with resolution of 16 micra, in opaque MED620 material). 

For generation of the reference model, the malocclusion incorporated was virtually treated, with the Ortho Analyzer software (3Shape; Copenhagen, Denmark). For this purpose, the library of the program was used to select the same brand and prescription of brackets that would afterwards be used on the intervention models: metal brackets, slot 0.022 x 0.028-in, pre-adjusted, MBT prescription, Mini Master series (American Orthodontics^®^, Sheboygan, USA) and simple pre-adjusted tubes, MBT prescription, of Ifit Non Convertible Buccal series (American Orthodontics^®^, Sheboygan, USA). Initially the setup was made, followed by the virtual positioning of the accessories in the ideal arch with stainless steel 0.021 × 0.025-in archwire, with the purpose of simulating the respective post-treatment positions. After this, the configuration of the final positions obtained was reverted to the malocclusion under study.

The intervention models (direct bonding and virtual bonding) were equally distributed, into two distinct time intervals, among 10 orthodontists, consisting of Group I (direct bonding of orthodontic accessories) and Group II (virtual bonding of orthodontic accessories). 

The direct bonding procedures were performed in pre-clinical conditions. The 10 sets of solid models were individually coupled to the head of the mannequins, with the vestibular surfaces already prepared (cleaned with 70% alcohol, followed by application and polymerization of a thin layer of adhesive (Transbond XT, 3M Unitek, Dental Products, St Paul, MN, USA). Each participant performed the bonding with free prescription, workflow and time, and on the bench there were clinical instruments and instruments for measuring the position of brackets. They performed the procedures with composite resin (Transbond XT, 3M Dental Products, St Paul, MN, USA), light-cured with LED light for 20 seconds, on each tooth, in both arches. 

Fifteen days later, these same operators performed virtual bonding. In the time interval between the two bonding procedures, 3Shape’s institutional video about virtual bonding was sent to each participant by e-mail. In addition, a written explanatory text about how the interaction with the virtual bonding software would occur and about the conditions of the models were presented shortly before they performed bonding. The bonding protocol was standardized since all the accessories were previously distributed at the midpoint of the facial axis of the clinical crown (FA point), on the vestibular surface of all the teeth - both maxillary and mandibular -, performed automatically by the program. Furthermore, all the participants used the same computer to perform the bonding procedure. All communication was done by web, via Skype, with interaction between the operator of the software and the participant, maintained strictly anonymous throughout the entire process. On one hand, an orthodontist from the specialized laboratory manipulated the software; and in the pre-clinic laboratory, each participant, emitting only a voice command, determined the definitive position of the accessory, according to his/her perception of ideal bonding. During all the bonding procedures, the researcher remained present, in the condition of observer. 

**Figure 1:**
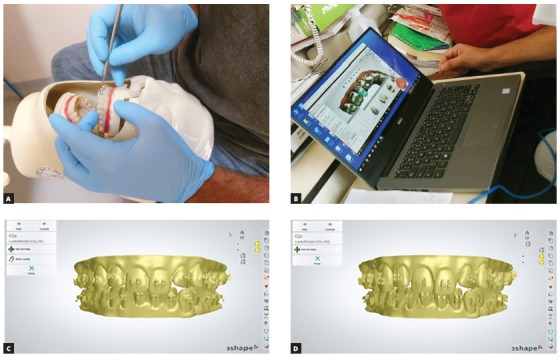
Presentation of models: A) solid model during direct bonding procedure; B) digital model during virtual bonding procedure; C) digital model after direct bonding procedure; D) digital model after virtual bonding.

Once the interventions were concluded, the solid model was scanned (intra-oral scanner, Scanner 3D, Ultrafast Optical Sectioning™, Trios^®^ Orthodontic) to make up the final sample, totaling 20 sets of digital models (stl format) (Fig 1). After this, the researcher measured the positions of the brackets by superimposing the 3D image (3Shape, module Appliance Designer 2017). Before performing the measurements, the file with the digital models was re-codified (20 random numbers were generated using the website randomization.com) and the decodification was sealed in an envelope. 

The ideal bonding position was defined by the absence of discrepancy in the position of each accessory, in the three dimensions (vertical [height], horizontal [mesiodistal] and angular [angulation]), in comparison with the virtual bond of reference validated for the study. 

» Deviation in the vertical component: distance projected, in millimeters, between the central inter-slot points, when the plane of visualization resulted from the transverse section along the central vertical axis of the reference accessory; displacements towards the gingival region were positive, and towards the incisal/occlusal region, negative. 

» Deviation in the horizontal component: distance projected, in millimeters, between the central inter-slot points, when the plane of visualization resulted from the transverse section along the central horizontal axis of the reference accessory; displacements towards the mesial region were positive, and towards the distal region, negative. 

**Figure 2:**
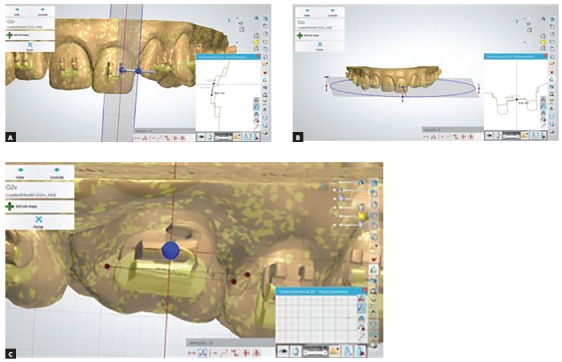
Measurement of bracket position by superimposition of 3D models: A) transverse section at the vertical axis of tooth #21 and the two-dimensional projection of the respective bracket heights; B) transverse section at the horizontal axis of tooth #21 and the two-dimensional projection of the mesiodistal positions of the respective brackets; C) transverse section in the frontal plane of tooth #16 with right side view of the angles of the respective simple tubes.

» Deviation in the angular component: the direct measurement, in angle, when the transverse section passed through the base of the reference bracket and its slot assumed angulation zero in relation to the frontal plane of visualization; when read from the mesial portion of the accessory, displacements in the anti-clockwise direction were positive and in the clockwise direction, negative (Fig 2). 

### Statistical analysis

After the data of the superimpositions were entered into the Excel 2013^®^ program (Microsoft Corp^®^, Redmond, USA), the respective identifications of the intervention models were re-codified. After this, the statistical analyses were performed using the Statistical Package for the Social Sciences (SPSS software for Windows, version 22.0, IBM Inc, Amonk, NY, USA).

Initially, the data were treated in a descriptive manner based on original values. Due to the importance of recognizing the nature of the mean error, and to differentiate the nature of its correction, analyses were performed considering the measurement of pure error. That is to say, the lower the value of the measurement, the smallest the error was; and negative or positive measurements represented the nature of this error. The Student’s-t test for paired samples was used for the purpose of comparing the difference in errors between the methods. In this case, the null hypothesis used was and the alternative hypothesis was , in which it was the mean of the results obtained by the manual method and by the virtual method. 

In addition, the groups of ranges were evaluated, accepting the limits of deviation of 0.5 mm for the linear dimensions (height and mesiodistal position) and of 1° for angulation, by using the Chi-square test. The frequencies of errors that exceeded the clinical limit in the groups of the direct procedure and virtual bonding were compared to measure the prevalence of errors in the positioning of accessories.^9^

All the results were considered significant for a level of significance of 5% (*p*< 0.05). 

## RESULTS

The distribution of the position of bonding the accessories to the groups of teeth, types of bonding, and descriptive measurements (mean, standard deviation), based on the original values of the deviations, are presented in Table 1. In total, 1654 brackets positions were analyzed (547 accessories - 10 solid models and 10 digital models), with 277, 276 and 274 resulting from direct procedures, in the vertical, horizontal and mesiodistal dimensions, respectively; and 277, 276 and 274 resulting from virtual bonding, in the vertical, horizontal and mesiodistal dimensions, respectively. Thirteen accessories failed and were excluded from the study (4 due to readout error after scanning, and the others due to debonding after direct bonding). 

**Table 1:** Distribution of the accessories in the groups of teeth and descriptive measurements of the original data, showing the deviations from ideal bonding, in the direct and virtual bonding procedures

Type of tooth	Direct bonding			Virtual bonding		
	Vertical (mm)	Horizontal (mm)	Angular (degrees)	Vertical (mm)	Horizontal (mm)	Angular (degrees)
Incisor	-0.651 ± 0.568^b.c^	0.118 ± 0.349	0.939 ± 3.446	-0.231 ± 0.483	0.097 ± 0.198	0.291 ± 2.207
Canine	-0.403 ± 0.863	0.166 ± 0.384	2.368 ± 4.930	-0.059 ± 0.538	0.167 ± 0.318	2.499 ± 4.696
Premolar	-1.081 ± 0.730	-0.133 ± 0.480	0.046 ± 4.906	-0.460 ± 0.663	-0.016 ± 0.385	0.526 ± 3.073
Molar	-0.902 ± 0.736	-0.116 ± 0.662	-1.295 ± 4.423	-0.384 ± 0.474	0.192 ± 0.425	-2.365 ± 5.396
Total	-0.813 ± 0.744	-0.013 ± 0.510	0.224 ± 4.544	-0.317 ± 0.560	0.102 ± 0.354	-0.110 ± 4.253

Note: the negative values indicate that the deviation of the orthodontic accessory, in comparison with ideal bonding, was more toward the distal direction (in the horizontal dimension); toward the occlusal/incisal direction (in the vertical dimension), or that the mesial portion of the accessory rotated towards the occlusal/incisal direction (in the angular dimension).

Comparative analysis between the types of bonding, considering the respective deviations in comparison with the ideal position, showed that the general mean, in the vertical dimension was 0.58 mm and 0.49 mm; in the horizontal, 0.33 mm and 0.24 mm; and in the angular, 3.18 and 2.89 degrees, for the direct and virtual procedures, respectively (Table 2). 

**Table 2:** Comparative analysis between direct and virtual bonding with regard to error, considering each dimension evaluated.

Dimension	Bonding	Descriptive measurements									p
		N	Minimum	Maximum	P_25_	Median	P_75_	Mean	Dp	CV	
Vertical (mm)	Direct	277	0.00	2.75	0.46	0.87	0.94	0.58	0.62	1.07	< 0.001
	Virtual	277	0.00	1.82	0.18	0.37	0.71	0.49	0.42	0.86	
	Direct - Virtual	277	-0.94	1.82	0.09	0.45	0.74	0.41	0.47	1.15	
Horizontal (mm)	Direct	276	0.00	2.57	0.16	0.32	0.39	0.33	0.83	2.52	< 0.001
	Virtual	276	0.00	1.18	0.09	0.23	0.28	0.24	0.84	3.50	
	Direct - Virtual	276	-0.79	2.20	-0.08	0.09	0.26	0.11	0.35	3.18	
Angular (degrees)	Direct	274	0.00	16.00	0.00	2.75	3.24	3.18	0.98	0.31	0.571
	Virtual	274	0.00	12.30	0.68	2.40	3.11	2.89	0.93	0.32	
	Direct - Virtual	274	-11.30	10.80	-2.40	0.00	2.70	0.13	3.85	29.62	

Note: the probability of significance refers to the Student’s-t test for paired samples.

There were significant differences between the two bonding methods in the vertical and horizontal dimensions. Relative to the distribution of errors, there was predominance in the direct bonding method, because the mean of differences between the two methods was positive. In the angular dimension, no significant difference was observed between the methods.

When comparing the bonding methods regarding the accuracy in the limits of deviation, 0.5 mm in the linear components, and 1 degree for angulation, there were significant differences in the three evaluated dimensions (Table 3). 

**Table 3:** Comparative analysis between virtual and direct bonding, when the limit of deviation was 0.5 mm for the linear dimensions and 1° for angulation, in the dimensions evaluated.

Dimension	Bonding	Accuracy		Total	p
		Yes	No		
Vertical (mm)	Direct	75 (27.1%)	202 (72.9%)	277	< 0.001
	Virtual	179 (64.6%)	98 (35.4%)	277	
Horizontal (mm)	Direct	202 (73.2%)	74 (26.8%)	276	0.004
	Virtual	230 (83.3%)	46 (16.7%)	276	
Angular (degrees)	Direct	98 (35.8%)	176 (64.2%)	274	0.044
	Virtual	76 (27.7%)	198 (72.3%)	274	

Note: The probability of significance is with reference to the Chi-square test.

In the vertical dimension, accuracy was observed in 64.6% of the teeth for the virtual bonding method; while in the direct bonding method, there was no accuracy in the majority of the teeth (72.9%). For the horizontal dimension, the percentage of accurately bonded teeth corresponded to the majority for the two methods, however, with a lower percentage for the direct bonding method. For the angular dimension, accuracy was observed in fewer than 40% of the teeth in both methods, however, in the direct bonding method, 35.8% of the teeth showed accuracy, and in the virtual bonding method, this percentage was even lower (27.7%).

## DISCUSSION 

The use of absolute values to demonstrate characteristics of the errors of accessories’ positions has been pointed out in the literature as a procedure that generates great discrepancy in the results.^5^ To eliminate this tendency, in this study, the Student’s *t* test for paired samples was used, in which the measurement evaluated was the difference in errors between the methods for each tooth. 

Regarding the direct bonding procedure, the general mean value of the deviations, expressed in absolute values, was 0.38 mm (height), 0.33 mm (mesiodistal position) and 3.18 degrees (angulation), with the optional use of instruments for measuring the position. In a previous study, with mandatory use of these instruments, bonding errors were also recorded in all dimensions, and the results found were 0.43 mm, 0.41 mm and 3.76 degrees, for height, mesiodistal position and angulation, respectively.^5^ In general, these measurements ranged between 0.34 mm and 0.43 mm, for the virtual discrepancies; between 0.19 mm and 0.41 mm, in the mesiodistal dimension, and between 2.57 and 5.54 degrees, for the angulations.^4^ ^-^ ^16^ It is consensus in the literature that the intraexaminer and interexaminers variability in perception of the ideal bonding position makes it unfeasible to achieve accuracy by means of the traditional methods.^14,17^ Thus, investigations have been directed towards refining the indirect bonding protocols.

In this context, the virtual bonding software has increasingly gained attention. Furthermore, the possibility of virtual measurement of the positioning of orthodontic accessories before the expression of the pre-adjusted appliances makes it easier to foresee possible bonding errors, and to guide early interventions, thus preventing the progression of undesirable orthodontic movements. However, the investigations converge on indirect evaluation of the quality of assembling the appliance in the subsequent stage, by quantifying the post-treatment clinical benefits.^9^ ^-^ ^18^ No studies about the accuracy of virtual bonding prior to guided bonding were found. Similarly, we did not find any comparative studies related to the direct bonding procedure. 

In our findings, positioning the accessory virtually represented an improvement in accuracy of the vertical dimension, in comparison with the direct procedure; resulting in almost twice the gain when the clinical limits were considered.

At any rate, the common divergences relative to the ideal positioning in the vertical dimension and the imprecisions in defining the height for each case individually and for each type of problem may be minimized with computer-aided bonding. Under these conditions, the changes required for each tooth movement are recorded, and this makes it feasible to conduct planning by means of multiple simulations of the result of treatment. The possibility of not positioning the accessories in accordance with the pre-established prescription, but in accordance with the needs of each patient individually, may optimize orthodontic practice. Further investigation of CAD/CAM orthodontic appliances is needed and ideally would require prospective randomized controlled trials.

Although this technology exists, it is still relatively expensive. Moreover, feasible solutions and potential improvements in the virtual bonding protocols depend on extensive cooperation among the orthodontists, laboratory professionals, dental schools and industries. For the purpose of greater therapeutic effectiveness and control, virtual Orthodontics is an irreversible step.^19^ ^-^ ^20^ Overcoming the learning curve, mainly of those concerned with the academic education of future orthodontists, will perhaps be another great challenge.

## CONCLUSION

When comparing the accuracy of the direct with the indirect virtual bonding procedures, considering the clinical limits of 0.5 mm deviation, for the linear components, and 1 degree for angulation, there were significant differences in the three dimensions evaluated: height (*p*< 0.001); mesiodistal position (*p*= 0,004) and angular (*p*= 0.044). Virtual bonding enabled a significant improvement in the vertical dimension, in comparison with the direct bonding procedure, with a percentage of correctness of 64.6% and 27.1%, respectively. The use of this resource could contribute to greater assertiveness in the positioning of orthodontic accessories during fixed appliance bonding, by means of a guided procedure. 
